# Content analysis of UK newspaper and online news representations of women's and men's ‘binge’ drinking: a challenge for communicating evidence-based messages about single-episodic drinking?

**DOI:** 10.1136/bmjopen-2016-013124

**Published:** 2016-12-23

**Authors:** C Patterson, C Emslie, O Mason, G Fergie, S Hilton

**Affiliations:** 1 MRC/CSO Social and Public Health Sciences Unit, University of Glasgow, Glasgow, UK; 2 Institute for Applied Health Research/School of Health and Life Sciences, Glasgow Caledonian University, Glasgow, UK; 3 Department of Geography, Durham University, Durham, UK

**Keywords:** PUBLIC HEALTH, QUALITATIVE RESEARCH, SOCIAL MEDICINE

## Abstract

**Objectives:**

In the UK, men's alcohol-related morbidity and mortality still greatly exceeds women's, despite an increase in women's alcohol consumption in recent decades. New UK alcohol guidelines introduce gender-neutral low-risk alcohol consumption guidance. This study explores how UK newspaper and online news represent women's and men's ‘binge’ drinking to identify opportunities to better align reporting of harmful drinking with evidence.

**Design:**

Quantitative and qualitative content analysis of 308 articles published in 7 UK national newspapers and the BBC News website between 1 January 2012 and 31 December 2013.

**Results:**

Articles associated women with ‘binge’ drinking more frequently than men, and presented women's drinking as more problematic. Men were more frequently characterised as violent or disorderly, while women were characterised as out of control, putting themselves in danger, harming their physical appearance and burdening men. Descriptions of female ‘binge’ drinkers' clothing and appearance were typically moralistic.

**Conclusions:**

The UK news media's disproportionate focus on women's ‘binge’ drinking is at odds with epidemiological evidence, may reproduce harmful gender stereotypes and may obstruct public understandings of the gender-neutral weekly consumption limits in newly proposed alcohol guidelines. In order to better align reporting of harmful drinking with current evidence, public health advocates may engage with the media with a view to shifting media framing of ‘binge’ drinking away from specific groups (young people; women) and contexts (public drinking) and towards the health risks of specific drinking behaviours, which affect all groups regardless of context.

**Strengths and limitations of this study**

- Quantitative and qualitative content analysis of a large, comprehensive, 2-year sample of UK national newspaper news and online news about ‘binge’ drinking.
- The findings illustrate how media portrayals of ‘binge’ drinking could be harmful, and identify opportunities for these portrayals to be better aligned with evidence.
- Content analysis facilitates understandings of the messages being presented to the public, but cannot determine the extent to which audiences' understandings are influenced by media representations of specific issues.
- Some findings may have differed if the data analysed had gone beyond text articles, for example, by incorporating images, video and social media content.

## Introduction

While the gap between women's and men's excessive and harmful consumption of alcohol in the UK has narrowed in recent years,^1^ men still drink more than women,^2^ experience more drink-related health and social problems and face twice women's alcohol-related mortality (15.9–7.8 deaths per 100 000 population, respectively, in 2012).^2^ National guidelines have typically issued different alcohol consumption guidance for men and women, with women being advised to drink less than men, but the UK have recently joined Australia and Portugal^3^ ^4^ in issuing the same low-risk consumption guidance for men and women, drawing on evidence that the health risks posed to each gender are similar at low-risk levels of consumption.^5^ In light of this shift towards gender neutrality in alcohol consumption guidelines, it is timely to consider how gender differences in drinking behaviours are represented and perceived.

As with most health issues, popular perceptions of drinking are likely to be influenced by mass media representations,^6–8^ and, in turn, differences in representations of women's and men's drinking are heavily influenced by how societies regulate gender roles.^9^ Media content analysis cannot tell us if, and how, specific content influences audiences' understandings and behaviours, and content analysis findings must be considered with that inherent limitation in mind. Nonetheless, exploring media representations of men's and women's drinking behaviours allows us to examine how shared cultural values around alcohol are articulated and constructed,^10^ which might inform efforts to improve media representations, and therefore public understandings, of harmful drinking behaviours.^11^

Research illustrates a clear gender divide in media portrayals of drinking behaviours, with men's drinking normalised and women's problematised. Day *et al*^12^ found that UK newspapers frequently characterised women as departing from idealised notions of femininity in terms of appearance (eg, weight gain, deeper voices, loss of good looks) and motherhood (eg, reduced fertility and unborn children). They found that men were framed as violent, but partial responsibility for men's violence was attributed to women, who were framed as sexual predators invading traditionally male-dominated drinking environments. Conversely, Wood *et al*^13^ found that UK newspaper coverage of proposed minimum unit pricing (MUP) policies presented women as being at ‘risk of harm’ from male aggression. Lyons *et al*^14^ found that both young women's and young men's magazines framed binge drinking as normative, adult and professional, with young men's magazines associating men's drinking with traditional masculine images and deriding young women's drinking behaviours. Nicholls^15^ found that UK television and newspaper news associated men's drinking with violence (both as perpetrators and victims) and women's drunkenness with unfeminine and undignified behaviours, such as loss of consciousness or partial nudity. Similarly, Atkinson *et al*^16^ found that UK magazines targeted at teenagers depicted women's drinking as more problematic than men's, and portrayed women as ‘behaving like men’ in male spaces, sexualised and highly emotional.

As Nicholls^15^ found, ‘binge drinking’, also called single-episodic drinking, is a key focus of media representations of harmful drinking; “problem drinking is less commonly associated with dependence and more commonly associated with binge, harmful and hazardous drinking”. Herring *et al*^17^ describe binge drinking as a ‘confused concept’ that has historically been, and continues to be, defined inconsistently. They suggest that ‘binge’ drinking is currently portrayed as a youth issue, despite evidence that single-episodic drinking is performed by various age groups,^2^ highlighting a potential area of media misrepresentation of population drinking behaviours. Qualitative evidence indicates that young women in Scotland define ‘binge’ drinking in terms of types of behaviour rather than the quantity of alcohol consumed,^18^ which may present an obstacle to clearly and objectively defining ‘binge’ drinking. Mixed-method evidence suggests that both male and female students in England perceive binge drinking and public drunkenness as masculine behaviours.^19^ Two studies based on qualitative data from New Zealand have highlighted the role of alcohol-related behaviours in young people's gender identities. First, Willott and Lyons^20^ found that young men and women perceived consuming large quantities of alcohol in a single episode (among other behaviours) as a key performance of masculinity, and highlighted the identity negotiations undertaken by men who do not engage in these normative, masculine drinking behaviours. Second, Hutton *et al*^21^ examined the challenges that young women experience in curating social media personae that balance engagement in ‘the culture of intoxication’ (ref. 21, p. 88) with maintaining respectability. Given the primacy of binge drinking in UK media coverage of harmful alcohol use,^15^ binge drinking is an important lens through which to examine gendered representations of drinking behaviours.

This study comprises a comprehensive, mixed-methods content analysis of 2 years of UK media coverage of binge drinking, designed to contribute new insights into a growing body of literature about gendered media representations of alcohol, with a particular focus on binge drinking. Our aim is to improve understandings of UK newspaper and online news representations of women's and men's ‘binge’ drinking, focusing not on how ‘binge’ drinking is defined, but rather on how different types of drinking behaviour are gendered in media content where those behaviours are labelled as ‘binge’ drinking. While the limitations of media content analysis must be taken into account, the improved understandings produced by this research may help to inform efforts to better align media representations of harmful drinking with current evidence, which could in turn improve public understandings of the risks of single-episodic drinking.

## Methods

We selected seven highly circulated^22^ ^23^ UK national newspapers, including their Sunday counterparts, and the most-read exclusively online news website^24^ (table 1). The chosen newspaper publications represented three genres: quality, middle-market tabloid and tabloid. This typology helps to ensure a sample that represents diverse readerships in terms of age, social class and political alignment.^25^ Quality newspapers are those that were traditionally printed in broadsheet format, have predominantly middle-class audiences, are politically diverse and are serious in tone. Middle-market tabloids are printed in tabloid format and are less serious in tone than quality-genre newspapers, and have a predominantly older, middle-class, right-wing audience. Tabloids are less serious and typically more sensationalist than middle-market tabloids, are politically diverse and have predominantly working-class audiences.

**Table 1:** Summary of publications and articles in the sample

				Article format					
		All articles		Standard		Feature		Editorial	
Genre/medium	Publication	n	Per cent	n	Per cent	n	Per cent	n	Per cent
Quality (n=86)	Guardian/Observer	58	18.8	48	20.3	9	17.3	1	5.3
	Independent/Independent on Sunday	17	5.5	11	4.6	5	9.6	1	5.3
	Daily Telegraph/Sunday Telegraph	52	16.9	44	18.6	5	9.6	3	15.8
Middle-market tabloids (n=39)	Daily Mail/Mail on Sunday	54	17.5	33	13.9	18	34.6	3	15.8
	Express/Sunday Express	13	4.2	8	3.4	3	5.8	2	10.5
Tabloids (n=75)	Daily Mirror/Sunday Mirror	13	4.2	10	4.2	2	3.8	1	5.3
	The Sun/News of the World	62	20.1	46	19.4	9	17.3	7	36.8
Online (n=39)	BBC News website	39	12.7	37	15.6	1	1.9	1	5.3
	Total	308	100	237	100	52	100	19	100

We selected a search period of 1 January 2012 to 31 December 2013, during which alcohol was subject to heightened media interest due to debates in the UK and Scottish parliaments around MUP.^26^ Much of the media coverage of MUP was related to ‘binge’ drinking due to the UK Government's Alcohol Strategy^27^ overtly positioning ‘binge’ drinking as the primary target of MUP. Relevant newspaper articles were identified using the Nexis database including articles from online editions of the two of the selected publications (The Guardian and The Daily Mail) that are archived in Nexis. BBC News articles were identified using the search function on the BBC website. The search string identified articles that contain both three or more mentions of ‘binge’ and one or more mentions of ‘drink OR drinker OR drinkers OR drinking’. Duplicate articles were removed, and articles were manually excluded if they did not predominantly focus on binge drinking in the UK, or if they were not in the news, feature or editorial formats. Initial searches identified 537 articles, of which 308 met the inclusion criteria and were eligible for detailed coding and analysis.

To systematically and comprehensively code and analyse the media content, we used a mixed-methods content analysis. First, we used quantitative content analysis to measure the frequency of content within the articles across the whole sample, and second, we performed qualitative content analysis of the content of a subsample of articles for more in-depth, reflexive analysis.^28^ From this perspective, quantitative content analysis is concerned with measuring and analysing manifest content (the surface-level content of the articles, the coding of which does not require interpretation on the part of the coder such that it can be recorded relatively objectively), while qualitative content analysis is concerned with latent content (the underlying meanings of the text, as interpreted by coders in an inherently subjective process).^29^ The mixed-methods approach in this research comprised the following steps: constructing a coding frame; coding manifest content using the coding frame; establishing the reliability of the data collected and excluding unreliable data; analysing the quantitative data; identifying aspects of manifest content to examine further using qualitative analysis; and finally performing thematic analysis of the latent content of articles containing the manifest content of interest.

To construct a coding frame with which to code manifest content, SH and CE read randomly selected articles from the sample in batches of 20, recording potential thematic categories relevant to the research topic as they emerged. At the point where a batch of articles was read without any new categories emerging, the list of categories was deemed to have reached saturation. The collected thematic categories were grouped into three broad thematic categories: How is binge drinking described and which sections of the population are associated with this behaviour?; How are the drivers of the binge drinking described and who is to blame?; and What are the consequences of binge drinking? These categories were chosen by a combination of a priori knowledge of the research topic and understandings of the content of the sample that emerged from close reading of the articles.^26^ The final coding frame comprised the three broad thematic categories and their subcategories, as well as fields to record more routine details of articles, such as publication, article format and word count. In addition, coders recorded whether each article mentioned men and women, allowing articles to be divided into four gender categories: those that mention women exclusively, those that mention men exclusively, those that mention both men and women and those that mention neither men nor women explicitly. These codes enabled analysis of whether themes varied by the gender focus of articles.

To collect quantitative data, OM read each article in turn, using the coding frame to record whether each theme was present within its manifest content. To ensure consistency of coding, GF coded a random subsample of 39 articles (12.7%). Linearly weighted κ tests of inter-rater agreement between OM and GF were then performed on each variable across those 39 double-coded articles, and variables that returned a coefficient below 0.8 were discarded from the study to ensure that only variables with strong agreement were retained for analysis. The coding frame data were then entered into SPSS for analysis. Statistical procedures comprised: a simple linear regression examining the relationship between publication quarter and the count of articles published; χ^2^ tests of whether thematic variables varied by gender focus, publication genre or article format; and paired t-tests comparing the means of thematic variables. The threshold of statistical significance is set at 0.01 throughout to mitigate the risk of type 1 errors.

Following quantitative analysis, the thematic category of ‘harms to appearance’ was deemed noteworthy and suitable for qualitative coding and analysis. The decision to focus on that theme was informed by stark gender differences in content that emerged from the quantitative analysis, and an expectation that valuable understandings could be gained from deeper analysis. To analyse the latent content of the 46 articles that had been coded as mentioning ‘harms to appearance’, GF and CP employed a thematic analysis approach^30^ using NVivo V.10, closely reading each article to generate initial codes, which were then collated into potential themes. GF and CP collaborated closely to assure that the themes were defined clearly and that they worked across the 46 articles. The findings from the thematic analysis are presented in terms of two broad themes, using typical quotations to illustrate article content.

## Results

### Quantitative findings

**Figure 1:**
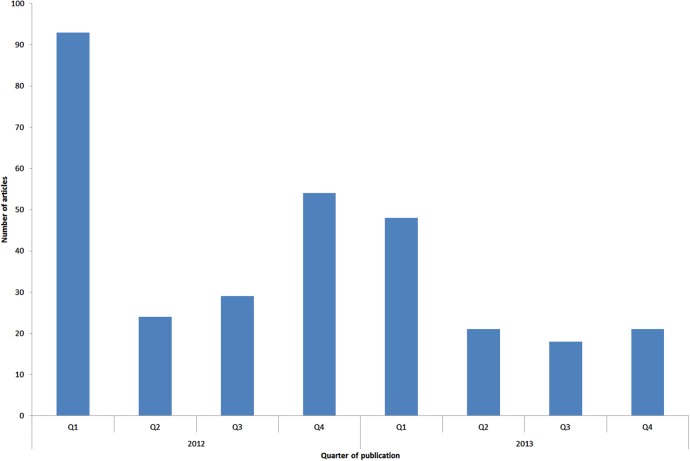
Frequency of publication of articles about ‘binge’ drinking by quarter.

Between 1 January 2012 and 31 December 2013, 308 articles about binge drinking were published in the seven print publications and one website included in the sample. Of these articles, 86 (27.9%) were published in ‘quality’ newspapers, 39 (12.7%) in middle-market tabloids, 75 (24.4%) in tabloids and 108 (35.1%) on BBC News (table 1). The majority of articles were standard news format (n=237, 76.9%), while 52 (16.9%) were feature articles and 19 (6.2%) editorials. The frequency of articles published per quarter decreased across the 2-year period (figure 1), but publication quarter was not a statistically significant predictor of article frequency (coefficient −6.714, p=0.088). There was a peak of 56 articles in March 2012, 66.1% of which were related to MUP, in particular the UK Prime Minister's announcement of plans to introduce MUP. There was elevated reporting from November 2012 to January 2013 (n=62), during which 53.2% of articles mentioned MUP, largely related to opposition to MUP within the UK Cabinet. In total, 133 (43.2%) articles mentioned MUP.

Articles were coded according to whether they mentioned binge drinking in women exclusively (n=68, 22.1%), men exclusively (n=30, 9.7%), both men and women (n=43, 14.0%), or neither men nor women explicitly (n=167, 54.2%). To be coded as mentioning both men and women, an article had to include specific discussion of each gender individually. There were no significant associations between gender category and either genre (p=0.382) or article format (p=0.303). Furthermore, neither publication genre nor article format was significantly associated with differences in reporting on any of the thematic categories listed in table 2.

**Table 2:** Social groups, locations and harms associated with binge drinking

	All articles (n=308)		Only females (n=68)		Only males (n=30)		Both (n=43)		Neither (n=167)		χ2 p Value (all)	χ2 p Value (Female vs male)
	n	Per cent	n	Per cent	n	Per cent	n	Per cent	n	Per cent		
Who is binge drinking?												
Mothers (including pregnant women)	32	10.4	23	7.5	0	0.0	9.0	2.9	0.0	0.0	0.000*	0.000*
Fathers	11	3.6	0	0.0	0	0.0	11.0	3.6	0.0	0.0	0.000*	–
Young people (including children and students)	119	38.6	24.0	35.3	8.0	26.7	18.0	41.9	69.0	41.3	0.419	0.401
Older people	17	5.5	5	1.6	0	0.0	3.0	1.0	9.0	2.9	0.499	0.127
Which locations of binge drinking are mentioned?												
At home	46	14.9	14	4.5	1	0.3	10.0	3.2	21.0	6.8	0.047	0.029
On city streets	60	19.5	12	3.9	5	1.6	10.0	3.2	33.0	10.7	0.087	0.906
Pubs, bars and clubs	56	18.2	12	3.9	5	1.6	6.0	1.9	33.0	10.7	0.836	0.906
What types of drink are associated with binge drinking?												
Wine	58	18.8	23	7.5	2	0.6	11.0	3.6	22.0	7.1	0.001*	0.004*
Spirits	58	18.8	22	7.1	12	3.9	4.0	1.3	20.0	6.5	0.000*	0.464
Beer	51	16.6	10	3.2	8	2.6	10.0	3.2	23.0	7.5	0.193	0.159
Cider	26	8.4	4	1.3	5	1.6	4.0	1.3	13.0	4.2	0.343	0.880
Alcopops	9	2.9	5	1.6	0	0.0	1.0	0.3	3.0	1.0	0.093	0.127
Shots	5	1.6	3	1.0	1	0.3	0.0	0.0	1.0	0.3	0.129	0.804
Cocktails	4	1.3	2	0.6	1	0.3	0.0	0.0	1.0	0.3	0.308	0.917
Fortified wine	4	1.3	1	0.3	0	0.0	2.0	0.6	1.0	0.3	0.186	0.504
Sparkling wine	3	1.0	1	0.3	0	0.0	1.0	0.3	1.0	0.3	0.676	0.504
Liqueurs	2	0.6	1	0.3	0	0.0	0.0	0.0	1.0	0.3	0.754	0.504
Lambrini	2	0.6	2	0.6	0	0.0	0.0	0.0	0.0	0.0	0.069	0.343
What harms of binge drinking are mentioned?												
Any personal harms	38	12.3	11	3.6	6	1.9	8	2.6	13	4.2	0.062	0.645
Harms to relationships	21	6.8	5	1.6	6	1.9	2	0.6	8	2.6	0.022	0.068
Harms to parenting	24	7.8	7	2.3	0	0.0	7	2.3	10	3.2	0.042	0.068
Any economic harms	107	34.7	16	5.2	8	2.6	17	5.5	66	21.4	0.080	0.739
Harms to the NHS	103	33.4	16	5.2	8	2.6	16	5.2	63	20.5	0.153	0.739
Harms to economic productivity	11	3.6	2	0.6	0	0.0	1	0.3	8	2.6	0.551	0.343
Any social harms	138	44.8	25	8.1	15	4.9	17	5.5	81	26.3	0.319	0.219
Social disorder and violence	128	41.6	19	6.2	14	4.5	16	5.2	79	25.6	0.044	0.071
Sexual assault and rape	19	6.2	9	2.9	4	1.3	2	0.6	4	1.3	0.005*	0.989
Fear	15	4.9	0	0.0	0	0.0	2	0.6	13	4.2	0.044	–
Any health harms	192	62.3	40	13.0	21	6.8	26	8.4	105	34.1	0.755	0.293
Death	77	25.0	8	2.6	16	5.2	10	3.2	43	14.0	0.000*	0.000*
Physical health	138	44.8	32	10.4	11	3.6	14	4.5	81	26.3	0.215	0.339
Harms to appearance	46	14.9	19	6.2	0	0.0	8	2.6	19	6.2	0.001*	0.001*

*Statistically significant at the 0.01 level.

NHS, National Health Service.

Articles were coded to identify the characteristics of binge drinkers and the specific drinks associated with binge drinking. Thirty-two (10.4%) of all 308 articles associated mothers (including pregnant women) with binge drinking, which differed significantly (p<0.000) from the 11 (3.6%) that mentioned fathers (table 2). There were no statistically significant relationships between gender focus and mentioning either younger or older people. While more articles that mentioned binge drinking in the home were focused exclusively on women, no drinking location was significantly associated with gender category (table 2). Wine (n=58, 18.8%) was the only type of drink significantly associated more with one gender than the other, being predominantly mentioned in articles focusing exclusively on women (p=0.004).

Almost two-thirds of articles (n=192, 62.3%) described health harms related to binge drinking, while one-quarter (n=77) mentioned risk of death. Mentions of specific categories of harm were recorded (table 2), and relationships between gender and specific harms were identified. Mentioning harms to appearance was significantly related to gender category (p=0.001); 19 of the 46 articles that mentioned harms to appearance mentioned women exclusively, while none of those 46 articles mentioned men exclusively. Harms to parenting were mentioned in 7 (10.3%) of the 68 articles that exclusively mentioned women, and none that exclusively mentioned men, but the difference was not significant (p=0.068). Sexual assault and rape were mentioned more frequently in articles exclusively mentioning women (n=9, 2.9%) than those exclusively mentioning men (n=4, 13.3%), but not significantly so (p=0.989). More articles directly associated the risk of death from drinking with men (n=16, 20.8%) than with women (n=8, 10.4%), and this difference was significant (p<0.000). Conversely, non-fatal physical health harms were associated with women (n=32, 23.2%) more frequently than men (n=11, 8.0%), albeit not significantly so (p=0.339).

### Qualitative findings

As identified in the quantitative findings, just 8 of the 46 articles that reported on ‘harms to appearance’ mentioned men, and none did so exclusively. These 46 articles were subjected to thematic analysis. A broad conceptualisation of ‘harms to appearance’ was used that included behavioural as well as aesthetic aspects of physical appearance that may be influenced (or perceived to be influenced) by single-episodic drinking. Using this broad definition, two key themes emerged from thematic analysis of the subsample: physical appearance and loss of self-control.

#### Physical appearance

Various harms to complexion were reported in relation to women, including the use of make-up to hide grey skin. Articles described specific damage to women's features, including hair, lips, noses and teeth, as well as using less specific descriptions of physical damage such as ‘ravaged’ (The Sun, 13 February 2012). An article headlined ‘Lambrini ruined my looks in 5 months’ (The Sun, 27 September 2013) associated an ‘attractive’ woman becoming ‘a swollen, haggard wreck’ with the consumption of a perry overtly marketed to young women. Weight gain caused by alcohol consumption was cited as both a product of alcohol use and a cause of ‘drunkorexia’, the practice of eating less to offset calories gained from alcoholic drinks.^31^ Drunkorexia was typically associated with women; one article stated that it ‘affects mainly young women’ (Daily Mail, 19 July 2012) while another defined it as a condition in which ‘calorie-conscious women skip meals in order to binge drink’ (Mirror, 20 December 2012).

Within the few articles in the subsample that mentioned men, male partial nudity was related to drunken behaviours, such as lost and ripped clothes, and intentional indecent exposure. Typifying this, the Daily Mail described a male student ‘flashing his penis in the street and laughing at his own loutish behaviour’ (19 June 2012). Conversely, partial nudity in women was more typically related to clothing choices presumably made while sober, with descriptions of revealing clothing, particularly miniskirts, and impractical shoes. One article described a fancy dress event in which ‘undergraduate girls take to the streets dressed in little more than their underwear’ (Daily Mail, 8 October 2012). Women were variously described as ‘scantily-clad’ (Daily Mail, 22 August 2012), ‘half-naked’ (The Sun, 3 March 2013) and ‘nearly bearing all’ (Daily Mail, 8 October 2012). Aside from choices of attire made in sobriety, harms to appearance that could befall women during drinking episodes included smeared makeup, vomit in hair, stained clothes and gravel embedded in knees. Articles also made mention of unintentional exposure of underwear or body parts by women, which was in contrast to men, who were typically described as exposed their genitalia deliberately.

#### Loss of self-control

Descriptions of women's and men's loss of physical control differed. Some articles depicted drunken women as burdening male partners. For example, two articles described women vomiting on their boyfriends, while one described female students needing ‘to be carried back to their rooms by boyfriends’ (Daily Mail, 19 June 2012). No articles characterised ‘binge’ drinking men as relying on, or burdening, their partners.

Women's aggressive behaviours were limited to verbal conflict, with drunken women characterised as noisy, argumentative, emotional and hard to control. An article describing Cardiff nightlife reported that drunken women ‘scream at their boyfriends in shop doorways’ and ‘sob in public’ (Daily Mail, 21 August 2012). Men were more typically associated with aggressive behaviours, including violent language and actions, damaging property and non-specific ‘loutish’ (Daily Mail, 19 June 2012) behaviour. While male aggression was presented as endangering others, women were characterised as putting themselves at risk, sometimes of undesirable sexual situations and assault. One article reproduced Office of National Statistics data about fines for drunk and disorderly behaviour, presenting men as much more likely than women to receive such fines, but suggesting that the number of women receiving the fines is increasing more rapidly than men (Telegraph, 14 June 2009).

## Discussion

We examined UK newspaper and online news representations of ‘binge’ drinking, exploring differences and similarities in representations of women's and men's drinking behaviours. Our quantitative analysis identified that women were associated with binge drinking more frequently than men, which conflicts with epidemiological evidence^2^ and young people's perceptions of ‘binge’ drinking as a masculine activity.^19^ We found various differences in how binge drinking was represented in association with different genders. Motherhood was mentioned in relation to ‘binge’ drinking more frequently than fatherhood. There were significant gender differences in the attribution of health harms, with male ‘binge’ drinking frequently associated with mortality and female ‘binge’ drinking more frequently associated with morbidity. Women's drinking was typically presented as more problematic than men's, and notably, harms to appearance were associated with women's ‘binge’ drinking much more frequently than men's. The articles in our sample associated wine with women's ‘binge’ drinking significantly more than men's, but our data did not identify significant gender associations with other specific types of drink.

Our qualitative analysis focused on depictions of the relationship between ‘binge’ drinking and appearance, comprising aspects of physical appearance and behaviour. Women engaged in ‘binge’ drinking were presented as helpless, physically incapacitated and transgressive, and as burdens to male partners, who were sometimes cast as carers for drunken women. ‘Binge’ drinking men were associated with physical and verbal aggression, but not behaviours that marked them as weak or vulnerable; while women were characterised as endangering themselves, men were more likely to endanger others. Articles typically depicted women as less able than men to maintain socially acceptable behaviour during single-episodic drinking. ‘Binge’ drinking was characterised as affecting women's physical appearance more than men's. Articles associated binge drinking with both immediate and long-term damage to physical characteristics and self-presentation. Descriptions of binge drinking women's dress had a moralistic tone that was absent in representations of men, with articles typically focusing on degrees of nudity and implicitly questioning the propriety of women's chosen attire. Our analysis suggests that media representations of women's ‘binge’ drinking do not focus solely on the effects of binge drinking on women, but also reflect broader social expectations about women's public behaviours.

Measham and Østergaard^32^ suggest that newspapers have constructed ‘binge’ drinking as a problem of young women adopting male behaviours:

The public face of binge drinking, […] a staple of early 21^st^ century tabloid newspapers, became the young woman emulating male consumption patterns […] with clothes askew stumbling around the city centre streets at night’.(p. 417)

There may be unintended consequences of this disproportionate focus on women's—as opposed to men's—single-episodic drinking in media reports. For example, it may reinforce harmful gender stereotypes by suggesting that drinking is more problematic for women than men, and may encourage victim blaming in relation to sexual assaults after drinking.^33^ It is notable that the media's disproportionately frequent association of women with single-episodic drinking in our sample is at odds with young adults' perceptions of these types of drinking behaviours as being masculine.^19^ ^20^ One potential explanation for this is that news producers regard women's ‘binge’ drinking as being of greater interest than men's because it is a deviation from gender norms. Whether disproportionate media focus on women's single-episodic drinking might influence a shift in public perceptions of how such behaviours correspond to gender identities may be a question for further research.

Our findings build on the growing body of literature about gendered media representations of alcohol by focusing specifically on portrayals of binge drinking. Depictions of women as unable to control themselves physically and emotionally echoed the findings of Atkinson *et al*,^16^ while the burden this weakness was depicted as placing on men could be seen as corroborating Day *et al*^12^ finding that traditionally male-dominated drinking environments and activities are perceived to be threatened by women's increasing involvement. Day *et als*^12^ finding that women were presented as making themselves vulnerable to male aggression was replicated in our finding that articles presented women as in danger, and men as dangerous. The depictions of women's dress and behaviours found in our analysis echoed Nicholls'^15^ findings. We found that whole or partial nudity in males was typically presented as frivolous or ridiculous, while women's partial nudity was presented with an underlying morally loaded tone, perhaps resulting from women being perceived as publically breaking social conventions.

Our analysis supports Herring *et al*'s^17^ observation that ‘binge’ drinking is popularly conceived in terms of specific social contexts, rather than being defined purely in terms of exceeding a certain quantity of alcohol in a limited time. Media framing of ‘binge’ drinking as an activity of young men and women, often in public spaces, disproportionately emphasises the context of single-episodic drinking and contributes to constructing binge drinking as something many (including older people or those who drink in private settings) might not associate themselves with. The disproportionate association of ‘binge’ drinking with young peoples' public, often antisocial (although performed within in social groups), drinking could have a damaging influence if it leads those who engage in single-episodic drinking outside of these contexts to misidentify their behaviours as harmless. The dominant narrative of ‘binge’ drinking may have become unhelpful as a health concept. As an alternative, it may be useful to draw a clear distinction in health communications between ‘binge drinking’, as an inconsistently defined, context-specific and value-laden term, and single-episodic drinking, as a specific, widespread and context-independent practice with a range of health consequences documented by research evidence.

Our findings have relevance to the development of alcohol guidelines. While the new UK guidelines propose gender-neutral low-risk consumption guidelines, the Department of Health acknowledges that men and women vary in their drinking behaviours and the long-term and short-term health risks they face.^5^ Media associations of men with alcohol-related mortality and women with alcohol-related morbidity are in line with epidemiological evidence.^5^ However, differences in how media coverage problematises women's and men's ‘binge’ drinking could promote perceptions of men's ‘binge’ drinking as less harmful than women's, potentially exacerbating men's harmful drinking behaviours and hindering public understandings and acceptance of the proposed guidelines.

Our conclusions are subject to limitations. While mass media's influence on public perceptions of health issues is extensively researched and well established,^6^ content analysis alone cannot determine the extent to which audiences' understandings correlate with media representations; audiences are subject to many influences beyond print and online news media, and do not consume media content in a passive, non-critical way. Studying other forms of media, such as television or social media, could help create a more complete understanding of media representations of ‘binge’ drinking. Furthermore, a larger sample size, achieved by choosing a longer timeframe or including more publications, could have increased the validity of our conclusions. A potential limitation of the data used is that the Nexis database stores articles in text-only format, omitting any images included in the original articles. Coders noted that text-only articles that appear factual and impartial could be perceived as more morally loaded with the original photographs included. Similarly, some articles may only reveal implicit gender bias when viewed with the original photographs included; this is illustrated in an article about ‘binge’ drinking on The Sun's website that contains seven images of women drinking and none of men, while the text makes no mention of gender.^34^ Finally, as the article search period concluded 2 years prior to the proposal of new alcohol guidelines, our analysis cannot tell us how representations changed immediately preceding, during or following the proposal. Further research might analyse how media coverage of alcohol guidelines represents ‘binge’ drinking and gender roles. Alternatively, comparative research could investigate whether media representations differed before and after the introduction of new guidelines. With these limitations taken into account, this comprehensive content analysis of a 2-year period of coverage contributes to understandings of gendered media representations of drinking, with a specific focus on ‘binge’ drinking.

In conclusion, our analysis suggests that popular representations of binge drinking may be harmful in three ways. First, if public audiences' understandings are influenced by media coverage of ‘binge’ drinking, associations of ‘binge’ drinking with certain demographics and situations could lead the public to underestimate the health risks of single-episodic drinking among those not typically depicted in ‘binge’ drinking narratives, particularly older people. Morally loaded representations of women's binge drinking may reproduce harmful stereotypes and stigma about the vulnerability of drunken women and the social unacceptability of female drunkenness. Finally, media content reinforcing a skewed representation of binge drinking may present a challenge to public health stakeholders seeking to promote evidence-based information, recommendations and policies with the goal of reducing alcohol-related health harms.

The misrepresentations identified by this research suggest that there may be a need for health advocates to engage with mass media to promote clear, evidence-informed messages about single-episodic drinking to help better align reporting on harmful drinking with evidence, with the ultimate goal of improving public understandings of harmful drinking behaviours. Media communication strategies may seek to avoid stereotypes of ‘binge drinking’ that implicitly define binge drinking in terms of social contexts and behaviours not directly related to alcohol consumption, instead offering clear, value-free definitions of single-episodic drinking based on specific quantities of alcohol and specific, time-bound episodes. Identifying the need to improve communication of single-episodic drinking is particularly relevant given the recent public consultation about new alcohol guidelines,^5^ and the UK Government's decision not to include specific guidance on single-occasion alcohol consumption within the final guidelines.
