# Correction

**DOI:** 10.1136/bmjopen-2016-013124corr1

**Published:** 2017-02-08

Patterson C, Emslie C, Mason O, *et al.* Content analysis of UK newspaper and online news representations of women's and men's ‘binge’ drinking: a challenge for communicating evidence-based messages about single-episodic drinking? *BMJ Open* 2016;**6:**e013124. doi: 10.1136/bmjopen-2016-013124

In Table 1, the figures in parentheses in the first column are incorrect. The corrected Table 1 is shown below.

**Table 1:** Summary of publications and articles in the sample

				Article format					
		All articles		Standard		Feature		Editorial	
Genre / medium	Publication	n	%	n	%	n	%	n	%
Quality (n=127)	Guardian / Observer	58	18.8	48	20.3	9	17.3	1	5.3
	Independent / Independent on Sunday	17	5.5	11	4.6	5	9.6	1	5.3
	Daily Telegraph / Sunday Telegraph	52	16.9	44	18.6	5	9.6	3	15.8
Middle-market tabloids (n=67)	Daily Mail / Mail on Sunday	54	17.5	33	13.9	18	34.6	3	15.8
	Express / Sunday Express	13	4.2	8	3.4	3	5.8	2	10.5
Tabloids (n=75)	Daily Mirror / Sunday Mirror	13	4.2	10	4.2	2	3.8	1	5.3
	The Sun / News of the World	62	20.1	46	19.4	9	17.3	7	36.8
Online (n=39)	BBC News website	39	12.7	37	15.6	1	1.9	1	5.3
	**Total:**	**308**	**100**	**237**	**100**	**52**	**100**	**19**	**100**

